# Stage 2: Who Are the Best Candidates for Robotic Gait Training Rehabilitation in Hemiparetic Stroke?

**DOI:** 10.3390/jcm10235715

**Published:** 2021-12-06

**Authors:** Wonjun Oh, Chanhee Park, Seungjun Oh, Sung (Joshua) H. You

**Affiliations:** 1 Department of Physical Therapy, Sports Movement Artificial Robotics Technology (SMART) Institute, Yonsei University, Wonju 26493, Korea; sdc01047@naver.com (W.O.); chaneesm@gmail.com (C.P.); 2 Department of Physical Therapy, Yonsei University, Wonju 26493, Korea; 3 Department of Physical Therapy, Kyungwoon University, Gumi 39160, Korea; seung-jun55@hanmail.net

**Keywords:** functional ambulation category, stroke, hemiplegia, robotic-assisted gait training, Walkbot

## Abstract

We aimed to compare the effects of robotic-assisted gait training (RAGT) in patients with FAC < 2 (low initial functional ambulation category [LFAC]) and FAC ≥ 2 (high initial functional ambulation category [HFAC]) on sensorimotor and spasticity, balance and trunk stability, the number of steps and walking distance in subacute hemiparetic stroke. Fifty-seven patients with subacute hemiparetic stroke (mean age, 63.86 ± 12.72 years; 23 women) were assigned to two groups. All patients received a 30-min Walkbot-assisted gait training session, 3 times/week, for 6 weeks. Clinical outcomes included scores obtained on the Fugl–Meyer Assessment (FMA) scale, Modified Ashworth Scale (MAS), Berg Balance Scale (BBS), trunk impairment scale (TIS), and the number of walking steps and walking distance. Analysis of covariance and analysis of variance were conducted at *p* < 0.05. Significant main effects of time in both groups on number of walking steps and distance (*p* < 0.05) were observed, but not in MAS (*p* *>* 0.05). Significant changes in FMA, BBS, and TIS scores between groups (*p* < 0.05) were observed. Significant main effects of time on BBS and TIS were demonstrated (*p <* 0.05). Our study shows that RAGT can maximize improvement in the functional score of FMA, BBS, TIS, steps, and distance during neurorehabilitation of subacute stroke patients regardless of their FAC level.

## 1. Introduction

Exoskeletal robotic-assisted gait training (RAGT) has rapidly gained popularity as a powerful and promising therapeutic modality to improve gait function in hemiparetic stroke; however, the best time to intervene and initial locomotor motor function level of patients for RAGT is unknown [1]. A report from the National Rehabilitation Center recently emphasized the need to assess the characteristics leading to the best effectiveness and optimal timing, intensity, and duration of post-stroke RAGT rehabilitation interventions [2]. Determining the patient’s initial ambulation level at which robot gait training is most effective remains problematic [3]. Therefore, there is a need to determine the best functional ambulatory category (FAC) for more effective and sustainable RAGT intervention outcomes in stroke patients. Morone proposed a conceptual scheme to classify the best robotic gait intervention types based on initial FAC in stroke robotic rehabilitation. Exoskeletal RAGT types, including the Lokomat (Hocoma, Zurich, Switzerland) and Walkbot (P&S Mechanics, Seoul, Korea), were suggested to be more effective in stroke patients with FAC < 2 (weight supporting group), whereas overground walking training was recommended for patients with FAC ≥ 2 (non-weight supporting group) [4].

An extensive systematic review of exoskeletal RAGT demonstrated that Lokomat and Walkbot have been commonly used to facilitate early mobilization using guidance mode (i.e., assist-as-needed) or impedance mode for stroke patients with FAC ≤ 2, showing varying results [5,6,7,8,9,10,11]. Specifically, a study on the Lokomat RAGT in subacute stroke patients showed positive improvements in gait function [12]. Walkbot RAGT studies consistently revealed positive improvements in balance and gait function in stroke patients [5,13,14,15]. Recent clinical studies compared functional balance and lower extremity function outcome measures between Locomat and Walkbot robotic gait training in individuals with non-ambulatory chronic acquired brain injury patients, demonstrating equivalent results.

To accommodate stroke patients with a wide range of FAC levels (0–4), we developed a Walkbot RAGT that offers (1) an interactive guidance mode; (2) progressive resistance force (isokinetic) mode, and (3) real-time feedback on ankle-knee-hip kinematics and kinetics based on task-oriented training being performed. Specifically, we believe that the Walkbot interactive guidance mode can assist in mobilizing the ankle-knee-hip joint to facilitate the reciprocal interlimb-coordinated locomotor pattern for stroke patients with FAC < 2, while progressive resistance force (isokinetic) mode strengthens weak ankle-knee-hip muscles for stroke patients with FAC ≥ 2. As these claims remain to be validated, the purpose of the present study was to determine the effects of Walkbot RAGT on sensorimotor recovery using the following outcomes: Fugl–Meyer Assessment (FMA) scale scores, spasticity based on the Modified Ashworth Scale (MAS), balance based on the Berg Balance Scale (BBS), and trunk stability based on the Trunk Impairment Scale (TIS), as well as the number of steps and walking distance in subacute stroke patients with FAC < 2 (low initial functional ambulation category [LFAC]) and FAC ≥ 2 (high initial functional ambulation category [HFAC]). We hypothesized that RAGT would produce equivalent improvements in sensorimotor recovery function based on FMA scale scores, spasticity based on MAS scores, balance based on BBS scores, and trunk stability based on TIS scores, as well as increased number of walking steps and walking distance in both groups.

## 2. Materials and Methods

### 2.1. Patients

In this retrospective study, we included patients who took part in the RAGT intervention as inpatients in the Department of Rehabilitation Medicine at Cheong Dam Hospital in Seoul, Korea between June 2017 and September 2019. Fifty-seven patients with subacute stroke (mean age, 63.86 ± 12.72 years; 23 women) were assigned to two groups. The experimental study protocol was approved by the Cheong Dam Hospital Institutional Review Board and Ethics Committee (IRB No. CDIRB-2021-005). Informed consent was obtained from all patients prior to participation. We included patients with subacute stroke who had been classified as FAC 0– 4 (FAC 0–1, [*n* = 30]; FAC 2–4, [*n* = 27]).

Inclusion criteria were as follows: (1) presence of subacute cortical/subcortical ischemic stroke; (2) age, 18–99 years; (3) FMA score, >2; (4) suitability for gait training as assessed clinically (ability to ambulate at least one step with a device/assistance); (5) height, 132–200 cm; (6) hip-knee joint length, 33–48 cm; and (7) length of knee joint to foot, 33–48 cm. Exclusion criteria were as follows: (1) cerebellar/brainstem stroke survivor; (2) body weight, >135 kg; (3) uncontrolled hypertension with blood pressure, >160/100 mmHg; (4) cardiopulmonary impairments affecting the ambulation test; (5) integumentary impairment, such as skin breakdown or bedsores around the suspension belt loading region, (6) significant and persistent mental illness, (7) lower extremity fixed contracture or deformity, (8) bone instability (non-consolidated fractures, unstable spinal column, or severe osteoporosis necessitating treatment with bisphosphonates); (9) other neurodegenerative disorders (amyotrophic lateral sclerosis or Parkinson’s disease); 1(0) MAS > 3 in the affected leg; (11) significant back or leg pain resulting in an inability to tolerate movement; and (12) decreased sensation impairing the ability to perceive whether the device was properly fitted.

### 2.2. Clinical Outcome Measures

#### 2.2.1. FAC

The FAC is assessed using the functional walking test, which evaluates ambulation ability. This 6-point scale assesses ambulation status by determining how much support from an assistant the patient requires when walking, regardless of whether they use a personal assistive device. The scoring scale ranges from 0 (non-ambulatory) to 5 (normal). Assistive devices such as orthoses were permitted if necessary. The FAC’s reliability and validity have been previously established [16].

#### 2.2.2. FMA Scale

The FMA scale is used to assess sensorimotor impairment in patients with hemiparetic stroke. It is an ordinal scale with 3 points for each item. A score of 0 is given for the item if the subject cannot perform the task. A score of 1 is given when the task is performed partially, and a score of 2 is given when the task is performed fully. However, reflex activity is measured using 2 points only, with a score of 0 (“cannot perform”) or 2 (“performs fully”) for the absence and presence of reflexes, respectively. The maximum total score that can be obtained on the FMA scale is 226, although it is common practice to assess all domains separately. The FMA’s reliability and validity have been previously established [17].

#### 2.2.3. MAS

The MAS is used to assess spasticity in patients with stroke, spinal cord injury, multiple sclerosis, cerebral palsy, traumatic brain injury, pediatric hypertonia, and central nervous system lesions. To obtain a score on the MAS, the patient’s limb is extended from a position of maximal possible flexion to maximal possible extension (the point at which soft resistance is met). Then, while moving from extension to flexion, the score is recorded using a scale ranging from 0 (no increase tone) to 4 (rigid in flexion or extension). The MAS’s reliability and validity have been previously established [18].

#### 2.2.4. BBS

The BBS is used to evaluate a patient’s ability to safely balance during a series of predetermined tasks. It is comprised of a 14-item list with each item scored on a five-point ordinal scale ranging from 0 (lowest level of function) to 4 (highest level of function). The BBS’s reliability and validity of the have been previously established [19].

#### 2.2.5. TIS

The TIS is used to assess motor impairment of the trunk after a stroke through the evaluation of static and dynamic sitting balance as well as coordination of trunk movement. Scoring ranges from 0 to 23. For each item, a 2-, 3-, or 4-point ordinal scale is used. On the static and dynamic sitting balance and coordination subscales, the maximum scores that can be attained are 7, 10, and 6 points. The TIS’s reliability and validity have been previously established [20].

#### 2.2.6. Number of Walking Steps and Walking Distance

Spatial and temporal characteristics of gait were measured using the Walkbot software system (P&S Mechanics, Seoul, Korea). The standard Walkbot system contains six sensor pads encapsulated in a rolled-up carpet with an active area of 3.66 m in length and 0.61 m in width. When the patient walked, footfalls were captured by the sensors as a function of time. The information was stored and analyzed offline to assess footfall patterns. The parameters evaluated were number of steps, distance and cadence. When both the feet are in contact with the heel, the number of steps were counted as +1. Walking distance was determined by multiplying the stride by the number of steps. The formula for cadence was dividing the stride by period and multiplying it by 3.6. The mean of three repetitions for each parameter was used in the analysis [21]. For the present study, gait steps and gait distance were used in the spatiotemporal domain.

### 2.3. Intervention

**Figure 1:**
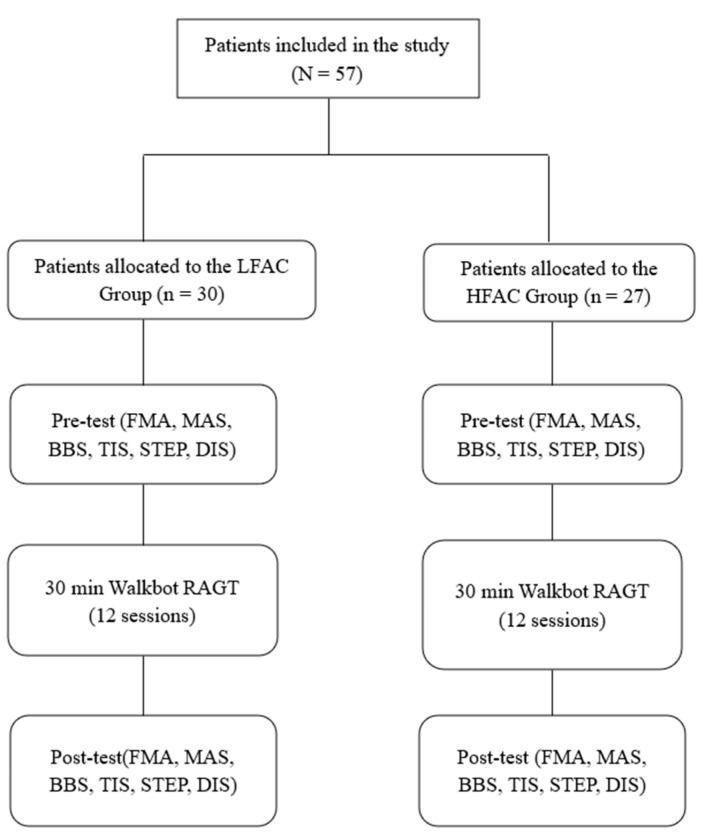
Flow chart.

**Figure 2:**
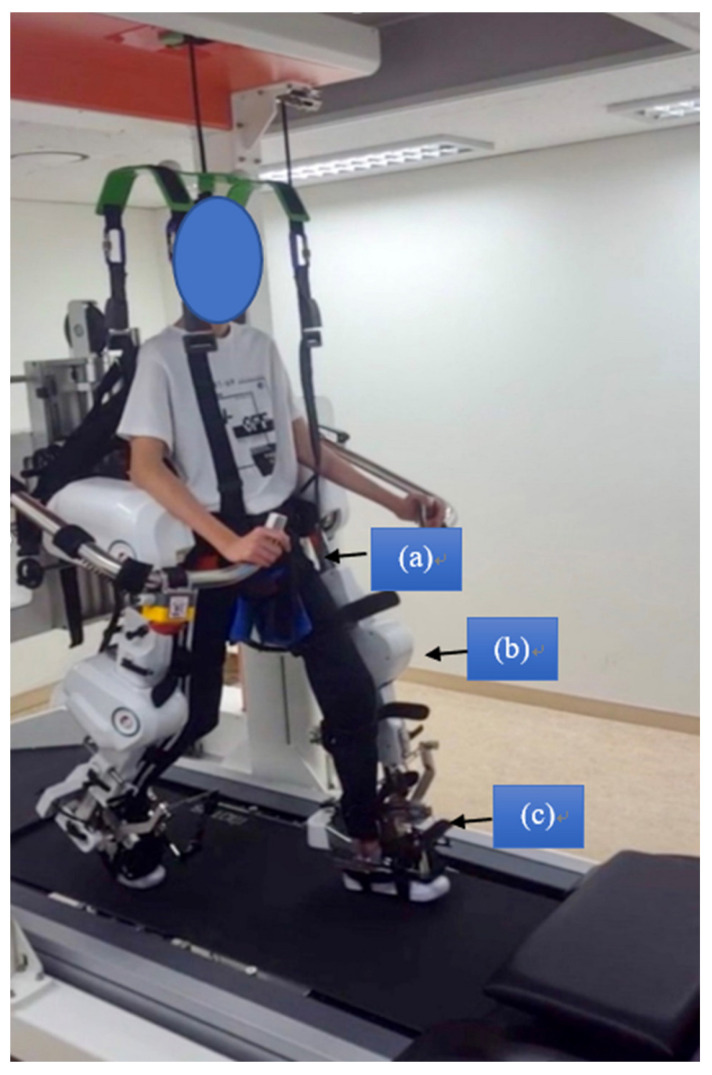
Walkbot exoskeletal system. (**a**) hip joint actuator including servomotor; (**b**) knee joint actuator including servomotor; and (**c**) ankle joint actuator including servomotor.

Both groups received the intervention three times/week every other day for six weeks (total: 18 sessions; minimum: 15 sessions) with a duration of 30 min (excluding set-up time) per session on the Walkbot-G system (Figure 1). Break time was provided whenever requested by the patient; however, the consecutive treatment time was maintained for at least 30 min. The Walkbot system is a robotic-assisted locomotor training device with built-in hip-knee-ankle inter-coordinated actuators to provide an optimal hip-knee-ankle-motion trajectory during locomotor training [22]. An adjustable leg length and control of ankle-joint range of motion enables the Walkbot system to accurately approximate human kinematics and kinetics [23]. This robotics system is designed to detect the patient’s gait characteristics in real-time (amount of participation or use in terms of active joint angular displacement excursion, active force/torque, and active weight-bearing center of pressure) and provides accurate and motivating real-time feedback concerning ankle-knee-hip kinematics and kinetics. Specifically, Walkbot RAGT can provide accurate proprioceptive, kinematic, and kinetic guidance, as well as variable error practice and high-intensity, repetitive, task-specific, and interactive exercises of the paretic lower limb [5]. Initially, the RAGT body weight support (BWS) was set at 100%, which was gradually decreased until the knees started to collapse into flexion during the stance phase. The physical therapist monitored the knee condition and controlled the BWS throughout the sessions. Walking speed was initially set at 0.5 km/h and gradually increased until the patients self-selected a comfortable speed. The guidance force was maintained at 100% during training. An expert physical therapist certified in robotic gait training provided all training procedures and verbal encouragement during the sessions (Figure 2).

In addition to the Walkbot RAGT, both HFAC and LFAC groups consistently received conventional physical therapy (CPT), twice a day, 30 min per session. CPT was based on neurodevelopmental treatment (NDT) which includes mobility and stability exercises, ROM exercise, stretching and strengthening to futher enhance neurodevelopment. The investigators who performed all the evaluations were blinded to the interventions administered to both groups to minimize the experimenter bias where the physical therapists who provided the interventions did not involve in the assessments. 

### 2.4. Statistical Analysis

Results are expressed as mean and standard deviation. A power analysis using G-Power software was conducted to assess the minimum sample size requirement, based on a prior pilot study. Based on a previous study, the sample size was determined to be 56 based on the effect size (eta squared, *η*^2^ = 1.02) and power (1 − *β* = 0.8) on FAC [12]. All continuous variables were analyzed using the Kolmogorov-Smirnov test, assuming a normal distribution. The study participants were divided into two groups based on their functional ambulation categories: LFAC (<2) and HFAC (≥2) groups. ANCOVA was used to test factor 1 (pretest and post-test effect in the HFAC-RAGT and LFAC-RAGT) and factor 2 (FAC scores) on all assessment parameters (FMA, BBS, MAS, TIS, STEP, and DIS). Post hoc comparisons were performed using a two-sample *t*-test. Additionally, minimal clinically important difference (MCID) was determined to estimate clinical meaningfulness and revealed that all outcome variables were clinically relevant in within-group changes. SPSS for Windows (version 25.0, SPSS, Chicago, IL, USA) was used to conduct statistical analyses. An alpha level was set at 0.05. Data were adjusted for imputative outliers and missing data.

## 3. Results

All patients who successfully completed the pre-test, intervention (at least 15 of 18 sessions), and post-test were included in the analysis. Table 1 summarizes patient demographic and clinical characteristics. There were no significant differences in baseline age, height, weight, type of stroke, and side of hemiplegia distribution variables between the LFAC and HFAC groups (Table 1). Baseline MAS, gait steps, and distance data were not significantly different, suggesting homogeneity of the groups (Table 2). Baseline FMA, BBS, and TIS parameters were statistically different between groups. No safety issues were reported, and no patients experienced any side effects associated with RAGT.

### 3.1. FMA

ANCOVA test indicates the FMA score is significantly different between the LFAC group and HFAC group (*p* = 0.000) (Table 3). Post hoc analysis confirmed that the intervention-related sensorimotor recovery was greater in the HFAC than LFAC (*p* = 0.000). However, the main effect of time was not a significantly different.

### 3.2. MAS

ANCOVA analysis indicates that the MAS are not significantly different between LFAC group and HFAC group, and time/Walkbot RAGT does not affect the MAS (*p* = 0.816) (Table 3).

### 3.3. BBS

ANCOVA demonstrated that RAGT intervention significantly changed the main effect of time of BBS/balance in both the LFAC and HFAC groups (*p* = 0.000). The BBS scores/balance levels were significantly different between the LFAC and HFAC groups (*p* = 0.000). Post hoc analysis confirmed that the balance was greater in the HFAC than LFAC (*p* = 0.000).

### 3.4. TIS

ANCOVA results indicated that the RAGT intervention/time effect significantly changed the trunk stability/TIS scores in both the LFAC and HFAC groups (*p* = 0.026) and between the LFAC and HFAC groups (*p* = 0.000). In addition, Post hoc analysis confirmed that the trunk balance was greater in the HFAC than LFAC (*p* = 0.001).

### 3.5. Number of Steps

ANCOVA demonstrated significant main effects of time in both the LFAC and HFAC groups (*p* = 0.000), but no group main effect was observed (*p* = 0.482) (Table 3). In addition, post hoc analysis confirmed a main effect of time, but there were no significant differences between groups.

### 3.6. Walking Distance

ANCOVA showed a significant main effect of time in both the LFAC and HFAC groups (*p* = 0.000), but no group main effects were observed *(p* = 0.593) (Table 3). Furthermore, post hoc analysis confirmed a main effect of time, but there were no significant differences between groups.

## 4. Discussion

This study investigated the effect of RAGT on sensorimotor recovery function using the FMA scale, spasticity based on the MAS, balance based on the BBS, and trunk stability based on the TIS, as well as number of walking steps and walking distance in subacute stroke patients with low or high initial FAC. As anticipated, regardless of the baseline FAC, both groups demonstrated significant improvements in sensorimotor, spatiotemporal, loss of balance, and trunk stability function following Walkbot RAGT intervention. Most importantly, the current findings dispute the notion that exoskeletal RAGT using Walkbot can provide clinically meaningful changes in balance and gait function in subacute hemiparetic stroke patients with low or high initial FAC. To our best knowledge, there is no previous study on this in the literature, which makes it difficult to compare our outcome measure data in subacute stroke patients with low or high initial FAC.

The functional score improvement amount analysis of the FMA sensorimotor function demonstrated an improvement in the functional score in the HFAC group (3.78) compared to that in the LFAC group (2.77), suggesting that RAGT was more beneficial for those with high initial ambulation ability than for those with low initial ambulation ability. This finding was consistent with previous RAGT studies that assessed the FMA sensorimotor recovery outcome measure in hemiparetic stroke patients [11,24]. Kim and colleagues (2020) reported that RAGT-induced sensorimotor recovery in FMA was approximately 13.04% greater than that of conventional physical therapy in 30 subacute stroke patients [11,24]. A similar improvement in FMA (18.27%) was observed after 4-week RAGT compared to that of conventional physical therapy in 34 subacute hemiparetic patients [11,24]. Such sensorimotor recovery may be associated with contemporary neurophysiological evidence of locomotor task-related neuroplasticity. Recent neurophysiological motor evoked potential evidence confirmed a positive correlation between FMA score and corticospinal excitability in the affected side of the primary motor cortex (M1) after RAGT in 13 patients with hemiparetic stroke [25]. Similarly, functional near-infrared spectroscopy neuroimaging data revealed 10–20% meaningful improvement in sensorimotor cortex (SMC), premotor cortex, and supplementary motor area network activation during RAGT in 15 subacute hemiparetic patients [11]. Kim and colleagues (2020) further supported the occurrence of neuroplastic changes in the ipsilesional motor cortex areas following RAGT [11].

Static, dynamic balance, and trunk stability analyses showed significant time and group effects, indicating that those with high initial FAC showed greater improvement in the functional static and dynamic balance (11.71) and trunk stability (3.85) scores than those with low initial FAC dynamic balance (4.03) and trunk stability (0.90). This result is in line with previous RAGT studies demonstrating more improvements in BBS score after RAGT than after conventional physical therapy in hemiparetic stroke [26,27]. Park and colleagues (2020) reported that RAGT-induced balance recovery in BBS was approximately 28.90% greater than that of conventional physical therapy in 14 subacute stroke patients [26,27]. A remarkable enhancement in BBS score was shown with an increase from 6.6 to 26 after RAGT compared to that of conventional physical therapy in 14 subacute hemiparetic patients [26,27]. Concurrently, trunk stability analysis revealed a moderate improvement (6.57%), which is compatible with previous TIS enhancement with RAGT (12.75%) [28]. A possible rationale for such improvement may be that Walkbot RAGT allowed patients to regain trunk stability because they were required to maintain upright postural stability while actively reciprocating the upper and lower limbs during locomotor training. 

Spatiotemporal gait analysis showed a significant main effect of time in number of walking steps and walking distance in both the LFAC and HFAC groups (both *p* = 0.000). This result corroborates previous robotic gait training studies [29,30]. Dae-Hyouk Bang and Won-Seob Shin reported the positive effects of Lokomat RAGT on gait speed (*p* = 0.003), cadence (*p* = 0.002), step length (*p* = 0.004), and BBS score (*p* = 0.048) [29,30]. Bonnyaud and colleagues evaluated the effect of RAGT on gait velocity (*p* = 0.02), cadence (*p* = 0.04), and step length (*p* = 0.04) recovery in 15 patients with hemiparetic stroke [29,30]. Such spatiotemporal gait improvement may have resulted from the fact that Walkbot RAGT provided an ample number of accurate, repetitive practice with a progressive passive-guided interactive isokinetic practice mode based on the individual’s initial FAC and sensorimotor conditions. Diserens and colleagues suggested that the accurate, repeated practice of locomotor behavior using RAGT may have facilitated the long-term potentiation underpinning synaptic neural plasticity, unmasking of the underutilized neural circuits, or utilization of the alternative neuronal pathways (e.g., ipsilateral corticospinal tracts, supplementary motor areas, and premotor cortex areas) [31,32], which enhanced sustainable functional gait function recovery as evidenced by measurement of spatiotemporal gait. The RAGT was designed to improve these spatiotemporal functions and offers possible explanations for the outcomes in the present study’s LFAC and HFAC groups.

Interestingly, contradicting Morone’s schema, we found greater functional score improvement in FMA, BBS, and TIS scores in the HFAC group than in the LFAC group, indicating that RAGT was beneficial for both groups; however, the HFAC group appeared to be respond better. The long-term effects of HFAC group participation in RAGT appear to be greater than those that can be observed following LFAC group participation. Depending on the initial FAC, complex neurophysiological factors (mood, pain tolerance, and previous experiences), neurobiological changes (cerebral metabolic changes, substrate depletion, alterations in regional neurotransmitter levels, and cerebral temperature), central command activation (sense of effort), and peripheral factors (afferent signals and responses from the cardiopulmonary system) may influence sensorimotor recovery. It is expected that the LFAC group may have lower neurophysiological thresholds than the HFAC group. For example, it seems that although the onset or severity of fatigue may depend on the type, intensity, and duration of the RAGT regimen, the LFAC group tended to show an increased difficulty in maintaining a given exercise intensity than the HFAC group, as assessed by ratings of perceived exertion (RPE), as evidenced by our previous study on RAGT. For example, in the LFAC group, RPE increased from 14.57 to 15.14, whereas in the HFAC group, it decreased from 13.43 to 12 after RAGT or conventional locomotor training [26]. This finding suggests that RAGT can induce muscle fatigue and increase cardiopulmonary endurance, which are important determinants for the development of peripheral and central fatigues [26,33]. Under normal or near-normal initial FAC group conditions, high-intensity isokinetic exercise, as performed in RAGT, does not contribute to central or peripheral fatigue, which may not restrict the supply of oxygen to the brain; this mechanism is offset by the poor perfusion of oxygen. However, it is expected that when intensive RAGT exercise is performed in the LFAC group, peripheral fatigue is more likely to occur, which decreases the oxygen supply, resulting in central fatigue [34]. Specifically, a recent study on Walkbot RAGT demonstrated that the RAGT regimen under the partial BWS condition (50%) significantly increased peripheral muscle fatigue, as evidenced by the isokinetic torque (17–24%), work (18–29%), and power of the quadriceps and hamstring muscles when compared to those with the RAGT regimen under the full BWS condition (100%) [33]. Nevertheless, further research is warranted to ascertain RAGT-induced peripheral and central fatigue and their important underlying neurophysiological mechanisms in stroke patients.

**Figure 3:**
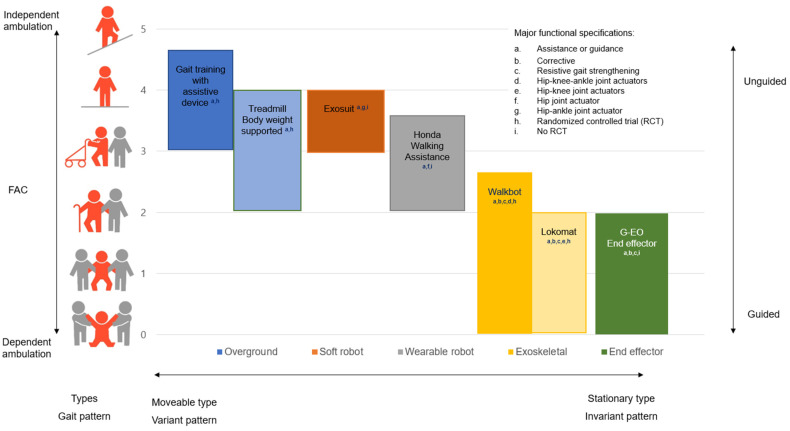
New robotic-assisted gait training schematic guideline based on baseline functional ambulatory category level.

Taken together with our results and collective clinical evidence in the current randomized controlled studies in RAGT, we have further developed a new RAGT schematic guideline (Figure 3) using the baseline FAC levels, which were initially conceptualized by Morone and colleagues. As illustrated in Figure 3, the RAGT schematic guideline is purported to provide robotic therapists with the appropriate clinical decision-making tools to select the optimal mode of locomotor rehabilitation robotics for the patient’s baseline ambulation level. Briefly, the RAGT schematic guideline is comprised of three core elements as follows: the patient’s baseline ambulation capacity, appropriate RAGT type, and amount of assistance provided (Figure 3). Baseline ambulation function is defined by FAC. Further, commercialized RAGTs can be categorized by type and gait pattern, from a “moveable type” or “more variant gait pattern” (overground treadmill gait, soft robot, and wearable robot) to a “stationary type” or “more invariant gait pattern” (exoskeletal robotics). An overground robotic device is based on the concept of assistance and facilitation of trunk control and lower limbs’ muscle activation pattern even in individuals with the low FAC levels (0–1), while RAGT aims at the mitigation of the abnormal synergistic gait via an intensive ankle-knee-hip interlimb locomotor coordinated training [35]. HFAC can perform intensive activities in ground-based robotic devices because of the aforementioned neurophysiological factors and neurobiological changes. In the case of static RAGT, this will be helpful for LFAC because it enables intensive training and can help the patient’s weight and muscle strength. However, our study also confirmed the improvement of HFAC in the Walkbot RAGT. Therefore, Figure 3 is as follows: The soft robot includes the Exosuit (Rewalk Robotics, Yokneam, Israel) [36], whereas wearable robots comprise the Honda Walking Assistance (Honda, Tokyo, Japan; hip control only) [37], Ekso bionics (Ekso bionics, Richmond, USA; knee-hip control) [38]. The “stationary” exoskeletal robotics include the Walkbot (ankle-knee-hip control), Lokomat (hip-knee control only), G-EO (Reha Tech AG, Olten, Switzerland; end-effector, foot control only) [39], Gait trainer (RehaStim, Berlin, Germany; end-effector, foot control only) [40], and gait-assistance robot (Toyota, Tokyo, Japan; hip-knee control only) [41]. The “amount of assistance or guidance” (unguided or partially guided) systematically varies depending on the patient’s ability to move the interlimb ankle-knee-hip joint within the predefined “ideal kinematic and kinetic locomotor trajectory” in a coordinated fashion. Importantly, additional research attempts should be made to develop appropriate RAGT modes and settings curtailed to an individual’s need and condition while considering several key factors (EMG motor control patterns of the lower limbs, gait analysis, neuropsychological elements—attention, cognitive function, etc.) when making the clinical decision for robotic stroke therapy.

A couple of study limitations should be considered in future investigations. A primary limitation is that although we extensively measured clinical sensorimotor recovery function, spasticity, balance, trunk stability, walking steps, and walking distance, the supporting mechanism for neuroplasticity changes was not investigated because of the lack of quantitative measuring equipment available for assessing neuroplasticity and robot-movement artifacts [42]. Future studies should use advanced imaging tools for measuring robotic training-induced neuroplasticity changes during and after RAGT in patients with hemiparetic stroke. Another limitation is the lack of follow-up evaluation, which can provide important information about the long-term effects of RAGT in post-stroke patients. Nevertheless, our study results indicate that Walkbot use consistently demonstrated advantages of RAGT on sensorimotor recovery, balance, trunk stability, number of walking steps, and walking distance in the LFAC and HFAC groups.

## 5. Conclusions

This clinical research study demonstrated functional score improved sensorimotor, balance, trunk stability, number of steps, and walking distance in LFAC and HFAC patients recovering from subacute stroke. The present results provide clinical evidence-based insights into the utilization of RAGT in patients with different initial FACs to maximize functional score improvement of sensorimotor and trunk stability, as well as balance, the number of steps, and walking distance functions in neurorehabilitation in subacute stroke survivors.

## Figures and Tables

**Table 1:** Demographic and clinical characteristics of patients (*N* = 57).

Characteristics	Total (*n* = 57)	LFAC (*n* = 30)	HFAC (*n* = 27)	*p*-Value
Age (years)	63.86 ± 12.72	65.47 ± 13.67	63.19 ± 11.8	0.708 ^1^
Height (cm)	164.63 ± 8.76	163.43 ± 9.48	165.96 ± 7.84	0.28 ^1^
Weight (kg)	62.46 ± 10.11	60.15 ± 8.37	65.02 ± 11.37	0.069 ^1^
Onset (month)	2.04 ± 3.06	2.63 ± 2.26	3.41 ± 3.54	0.345 ^1^
Gender				
Male (%)	34 (60%)	16 (53%)	18 (67%)	0.306 ^2^
Female (%)	23 (40%)	14 (47%)	9 (33%)	
Type of stroke				
Hemorrhage (%) Infarction (%)	33 (58%) 24 (42%)	19 (63%) 11 (37%)	14 (52%) 13 (48%)	0.381 ^2^
Side of hemiplegia				
Left (%)	36 (63%)	19 (64%)	17 (63%)	0.977 ^2^
Right (%)	21 (37%)	11 (36%)	10 (37%)	

Values are presented as the mean ± standard deviation. Abbreviations: LFAC, low initial functional ambulation category; HFAC, high initial functional ambulation category; denotes when *p*-value was less than 0.05. ^1^ *p*-value of the frequentist *t*-test; ^2^ *p*-value of the Chi-squared test of independence.

**Table 2:** Baseline clinical outcome measures characteristics of the patients (*N* = 57).

Pre-Test	LFAC	HFAC	*p*-Value
FMA	12.73 ± 16.15	32.59 ± 24.25	0.001 *
MAS	1.57 ± 0.82	1.37 ± 0.74	0.348
BBS	3.2 ± 3.46	15.48 ± 10.33	0.000 *
TIS	3.97 ± 5.33	9.11 ± 5.41	0.001 *
STEP	646.4 ± 347.67	654.15 ± 340.79	0.933
DIS	350.43 ± 185.58	364.74 ± 223.96	0.793

Data are presented as the mean ± standard deviation. Abbreviations: LFAC, low initial functional ambulation category; HFAC, high initial functional ambulation category; FMA, Fugl–Meyer assessment; MAS, Modified Ashworth scale; BBS, Berg Balance Scale; TIS, Trunk Impairment Scale; STEP, number of steps; DIS, walking distance. * *p*-value obtained by independent *t*-test.

**Table 3:** Clinical outcome data difference between *LFAC* and *HFAC* groups.

	LFAC			HFAC			*p*-Value		
	Pre-Test	Post-Test	Mean Change, MCID	Pre-Test	Post-Test	Mean Change, MCID	Time Main Effect	Between Groups	Time × Group
FMA	12.73 ± 16.15	15.5 ± 17.15	2.77 < 3.13	32.59 ± 24.25	36.37 ± 24.94	3.78 < 4.8	0.404	0.000 **	0.303
MAS	1.57 ± 0.82	1.47 ± 0.73	−0.1 < 0.13	1.37 ± 0.74	1.37 ± 0.74	0 < 0.14	0.805	0.363	0.000
BBS	3.2 ± 3.46	7.23 ± 4.6	4.03 ^‡^ > 0.84	15.48 ± 10.33	27.19 ± 6.25	11.71 ^‡^ > 1.2	0.000 **	0.000 **	0.000 **
TIS	3.97 ± 5.33	4.87 ± 5.59	0.9 < 1.02	9.11 ± 5.41	12.96 ± 5.26	3.85 ^‡^ > 1	0.026 **	0.000 **	0.167
STEP	646.4 ± 347.67	1043.83 ± 346	397.43 ^‡^ > 63.17	654.15 ± 340.79	1125.07 ± 311.58	470.92 ^‡^ > 59.96	0.000 **	0.482	0.000
DIS	350.43 ± 185.58	564 ± 183.85	213.57 ^‡^ > 33.57	364.74 ± 223.96	590.22 ± 216.55	225.48 ^‡^ > 41.67	0.000 **	0.593	0.000

Data are presented as the mean ± standard deviation. Abbreviations: LFAC, low initial functional ambulation category; HFAC, high initial functional ambulation category; FMA, Fugl–Meyer assessment; MAS, Modified Ashworth scale; BBS, Berg Balance Scale; TIS, Trunk Impairment Scale; STEP, number of steps; DIS, walking distance. ANOVA, analysis of variance ANCOVA, analysis of covariance ** *p* < 0.01. ^‡^ Change in Minimal Clinical Important Difference (MCID) is significant.

## Data Availability

The data presented in this study are available on request from the corresponding author.
